# Artificial Intelligence Algorithm for Subclinical Breast Cancer Detection

**DOI:** 10.1001/jamanetworkopen.2024.37402

**Published:** 2024-10-03

**Authors:** Jonas Gjesvik, Nataliia Moshina, Christoph I. Lee, Diana L. Miglioretti, Solveig Hofvind

**Affiliations:** 1 Cancer Registry of Norway, Norwegian Institute of Public Health, Oslo, Norway; 2 Department of Radiology, University of Washington School of Medicine, Seattle; 3 Fred Hutchinson Cancer Center, Seattle, Washington; 4 Division of Biostatistics, Department of Public Health Sciences, University of California, Davis; 5 Kaiser Permanente Washington Health Research Institute, Seattle; 6 Department of Health and Care Sciences, Faculty of Health Sciences, The Arctic University of Norway, Tromsø, Norway

## Abstract

**Importance:**

Early breast cancer detection is associated with lower morbidity and mortality.

**Objective:**

To examine whether a commercial artificial intelligence (AI) algorithm for breast cancer detection could estimate the development of future cancer.

**Design, Setting, and Participants:**

This retrospective cohort study of 116 495 women aged 50 to 69 years with no prior history of breast cancer before they underwent at least 3 consecutive biennial screening examinations used scores from an AI algorithm (INSIGHT MMG, version 1.1.7.2; Lunit Inc; used September 28, 2022, to April 5, 2023) for breast cancer detection and screening data from multiple, consecutive rounds of mammography performed from September 13, 2004, to December 21, 2018, at 9 breast centers in Norway. The statistical analyses were performed from September 2023 to August 2024.

**Exposure:**

Artificial intelligence algorithm score indicating suspicion for the presence of breast cancer. The algorithm provided a continuous cancer detection score for each examination ranging from 0 to 100, with increasing values indicating a higher likelihood of cancer being present on the current mammogram.

**Main Outcomes and Measures:**

Maximum AI algorithm score for cancer detection and absolute difference in score among breasts of women developing screening-detected cancer, women with interval cancer, and women who screened negative.

**Results:**

The mean (SD) age at the first study round was 58.5 (4.5) years for 1265 women with screening-detected cancer in the third round, 57.4 (4.6) years for 342 women with interval cancer after 3 negative screening rounds, and 56.4 (4.9) years for 116 495 women without breast cancer all 3 screening rounds. The mean (SD) absolute differences in AI scores among breasts of women developing screening-detected cancer were 21.3 (28.1) at the first study round, 30.7 (32.5) at the second study round, and 79.0 (28.9) at the third study round. The mean (SD) differences prior to interval cancer were 19.7 (27.0) at the first study round, 21.0 (27.7) at the second study round, and 34.0 (33.6) at the third study round. The mean (SD) differences among women who did not develop breast cancer were 9.9 (17.5) at the first study round, 9.6 (17.4) at the second study round, and 9.3 (17.3) at the third study round. Areas under the receiver operating characteristic curve for the absolute difference were 0.63 (95% CI, 0.61-0.65) at the first study round, 0.72 (95% CI, 0.71-0.74) at the second study round, and 0.96 (95% CI, 0.95-0.96) at the third study round for screening-detected cancer and 0.64 (95% CI, 0.61-0.67) at the first study round, 0.65 (95% CI, 0.62-0.68) at the second study round, and 0.77 (95% CI, 0.74-0.79) at the third study round for interval cancers.

**Conclusions and Relevance:**

In this retrospective cohort study of women undergoing screening mammography, mean absolute AI scores were higher for breasts developing vs not developing cancer 4 to 6 years before their eventual detection. These findings suggest that commercial AI algorithms developed for breast cancer detection may identify women at high risk of a future breast cancer, offering a pathway for personalized screening approaches that can lead to earlier cancer diagnosis.

## Key Points

**Question:**

Can commercial artificial intelligence (AI) tools for cancer detection on screening mammograms estimate the development of breast cancer years before diagnosis?

**Findings:**

In this cohort study of 116 495 women undergoing at least 3 consecutive rounds of biennial mammography screening, mean absolute differences in AI scores between breasts of women developing screening-detected cancer were 21.3, 30.7, and 79.0 at the first, second, and third study rounds, respectively. Mean absolute AI scores were higher for breasts developing vs not developing cancer 4 to 6 years before their eventual detection.

**Meaning:**

This study suggests that a breast-level AI score may be able to estimate the risk of future breast cancer and may be used to identify women at high risk who may benefit from preventive measures, including supplemental screening.

## Teaser

This cohort study examines whether a commercial artificial intelligence algorithm for breast cancer detection could estimate the development of cancer years before diagnosis.

## Introduction

Mammography screening reduces mortality from breast cancer^1,2,3^; however, its accuracy is imperfect.^2,4,5,6^ Various strategies to improve interpretive performance of mammography have been used for decades, including double reading.^2,5,7^ Recently, multiple commercial artificial intelligence (AI) algorithms have obtained regulatory approval as adjunct tools for radiologist interpretation, with promising results for detecting cancer present on mammograms.^5,8,9,10,11^

These AI algorithms have been developed to mark areas of concern and provide breast-level and examination-level malignant neoplasm scores to aid interpreting radiologists.^12,13,14^ However, emerging research suggests that these same AI scores may potentially detect imaging features associated with future breast cancers years before they are detected clinically.^14,15,16^ If the scores of commercial AI algorithms developed for immediate cancer detection can also estimate future cancer risk, then more accurate and reliable short-term risk estimation could lead to tailored, personalized preventive measures (eg, more frequent or supplemental imaging), possibly resulting in earlier breast cancer detection and less-aggressive treatment. Analyses of AI breast cancer detection scores for consecutive mammography screenings prior to diagnoses are needed to evaluate the potential for using these tools to estimate future risk of the disease.

In this study, we used AI cancer detection scores recorded on mammography from multiple consecutive screening rounds of a national screening program, BreastScreen Norway.^17^ We combined consecutive AI scores with long-term cancer outcomes to examine whether a regulatory-cleared, commercial AI algorithm for breast cancer detection could estimate the development of future breast cancers diagnosed on subsequent screening rounds.

## Methods

This population-based retrospective cohort study was approved by the Regional Committees for Medical and Health Research Ethics and had a legal basis in accordance with Articles 6 (1) (e) and 9 (2) (j) of the General Data Protection Regulation.^18^ The data were disclosed with legal bases in the Cancer Registry Regulations section 3-1 and the Personal Health Data Filing System Act section 19 a to 19 h.^19,20^ Written consent was waived under the Cancer Registry Regulation. This study was conducted following the Strengthening the Reporting of Observational Studies in Epidemiology (STROBE) reporting guideline for cohort studies.

The Cancer Registry of Norway administers the national screening program for breast cancer, BreastScreen Norway.^17^ The program offers 680 000 Norwegian women aged 50 to 69 years 2-view (craniocaudal [CC] and mediolateral oblique [MLO] projections) digital mammography screening every 2 years (24 ± 6 months).^17^ Radiologist assessments and breast cancer outcomes are prospectively recorded for all screening examinations. From 2017 to 2021, the screening attendance rate was 76%, the recall rate was 3.3%, the screening-detected cancer rate was 6.2 per 1000 screening examinations, and the interval cancer rate was 1.8 per 1000 screening examinations.

The Cancer Registry has an agreement with Lunit Inc for research use of their AI software.^21^ None of the authors are employed by or are consultants for Lunit. Some of the authors (J.G. and S.H.) had full control of the data, and all of the authors had full control of the information submitted for publication. Lunit Inc had no access to the data and were not involved in any part of the study.

### Study Setting and Data Sources

**Figure 1.**
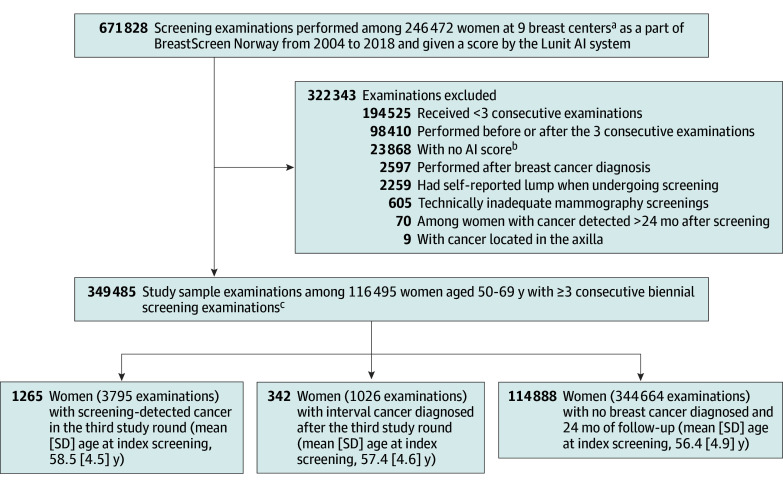
Study Sample With Exclusions AI indicates artificial intelligence. ^a^Agder, Hedmark, Oppland, Møre og Romsdal, Nordland, Troms and Finmark, Trøndelag, Vestre Viken, and Østfold. ^b^Missing AI information for 1 or more views. ^c^No breast cancer diagnosed before the third study round.

We obtained digital screening mammograms and associated clinical assessments and outcomes for examinations performed at 9 breast centers in BreastScreen Norway from September 13, 2004, to December 21, 2018 (Figure 1). All examinations were performed using Siemens Mammomat Inspiration (Siemens AG). All examinations performed in BreastScreen Norway are independently interpreted by 2 breast radiologists, and each radiologist assigns each breast a score from 1 to 5.^17^ A score of 1 indicates normal findings; 2, probably benign; 3, intermediate suspicion; 4, probably malignant neoplasm; and 5, high suspicion of malignant neoplasm. If either radiologist gives a score of 2 or higher, the examination is discussed in a consensus meeting to decide whether to recall the women for diagnostic evaluation.

We applied the AI algorithm (INSIGHT MMG, version 1.1.7.2; Lunit Inc; used September 28, 2022, to April 5, 2023) to all mammography screenings. INSIGHT MMG is a commercial AI algorithm with regulatory approval in Europe (CE [conformité européenne] marked) and the US (Food and Drug Administration cleared). The algorithm provided a continuous cancer detection score for each examination ranging from 0 to 100, with increasing values indicating a higher likelihood of cancer being present on the current mammogram.

### Study Population

A total of 246 472 women underwent 671 828 screening examinations during the study period (Figure 1). The study sample included women without a history of breast cancer who had at least 3 consecutive biennial screening rounds, where at least the first 2 rounds were not associated with a breast cancer diagnosis. Among women with a breast cancer diagnosis, data from the closest examination prior to the breast cancer diagnosis and the previous 2 examinations were included. Among women without screening-detected cancer, data from the first 3 consecutive screening examinations in BreastScreen Norway, as well as 2 additional years of follow-up, were included. The following examinations were excluded: those among women with fewer than 3 screening examinations (n = 194 525), those performed before and after the 3 consecutive examinations included in the study (n = 98 410), those without AI scores (n = 23 868), those performed after breast cancer diagnosis (n = 2597), those among women who reported a palpable lump at screening (n = 2259), technically inadequate examinations (n = 605), those among women with breast cancer detected more than 24 months after the third consecutive screening examination (n = 70), and those where the cancer was located in the axilla (n = 9). For women with bilateral cancer, the most malignant case (histologic type, tumor diameter, histologic grade) was included as the cancer case.

### Measures, Definitions, and Outcomes

A negative screening result was defined as an examination with an interpretation score of 1 on both breasts by both readers, those selected for consensus but determined negative (ie, no recall for further assessment), and examinations resulting in a recall for further assessment with no cancer diagnosed.

Breast cancer was defined as ductal carcinoma in situ or invasive breast carcinoma. All cancer cases were histologically verified.^17^ Reporting of cancer information to the Cancer Registry of Norway has been mandatory by law since 1953, and the completeness has been reported to be 98.8% for breast cancer.^22^

We defined the first of the 3 consecutive examinations as the first study round. The examination performed 2 years later was considered the second study round, and the examination 2 years after that was considered the third screening round. Screening-detected cancer was defined as breast cancer detected after a recall due to findings on the third study round examination, after negative results in study rounds 1 and 2. Interval cancers were detected within 24 months after the third study round mammography that was interpreted as negative or resulted in a negative diagnostic workup. Based on these definitions, we divided the study cohort into 3 groups: (1) women with screening-detected breast cancer on the third study screening round, (2) women with interval cancer diagnosed after the third study screening round, and (3) women with no breast cancer diagnosed after 3 consecutive examinations and 2 additional years (24 months) of follow-up (a total of 6 years without cancer diagnosis) (Figure 1).

An AI score for cancer detection was provided for each view (CC and MLO) of each breast. We used the highest score of the 2 views as the score for that breast. The highest score for all 4 images was used as the examination-level AI score.

### Statistical Analysis

Statistical analyses were performed from September 2023 to August 2024. We reported sample mean values of the AI score for each breast and absolute differences of the scores between the 2 breasts with SDs and median values with IQRs, separately for each study round for women with a diagnosis of screening-detected or interval cancer. For women with no breast cancer diagnosed during the entire study period, the same values averaged over both breasts were estimated for each of the 3 study rounds. Values by projection (CC and MLO) are reported in eTable 2 in Supplement 1. The percentage of examinations with a difference in AI score of 20 units or more between breasts that developed and breasts that did not develop breast cancer and for randomly chosen right and left breasts for those that did not develop breast cancer is shown in eTable 1 in Supplement 1.

We estimated cancer detection rates and false-positive rates associated with different thresholds for hypothetical triage scenarios using the AI score or the absolute difference in scores between breasts. We also evaluated how different hypothetical triage scenarios for AI score and absolute difference in scores between breasts could be associated with recall and cancer detection rates.

The area under the receiver operating characteristic curve (AUC) for the AI score and the absolute difference in scores between breasts for estimating screening-detected cancer vs no cancer, interval cancer vs no cancer, and screening-detected cancer and interval cancer vs no cancer were computed nonparametrically with 95% Bamber-Hanley CIs. We used Stata MP, version 18.0 (StataCorp LLC) for all statistical analyses and for generating descriptive figures.

## Results

Our final study cohort included data from 116 495 women who underwent at least 3 consecutive screening rounds in BreastScreen Norway, with a total of 1265 screening-detected cancers and 342 interval cancers (Figure 1). The mean (SD) patient age at the first study round was 58.5 (4.5) years for women with screening-detected cancer, 57.4 (4.6) years for women with interval cancer, and 56.4 (4.9) years for women without breast cancer.

**Figure 2.**
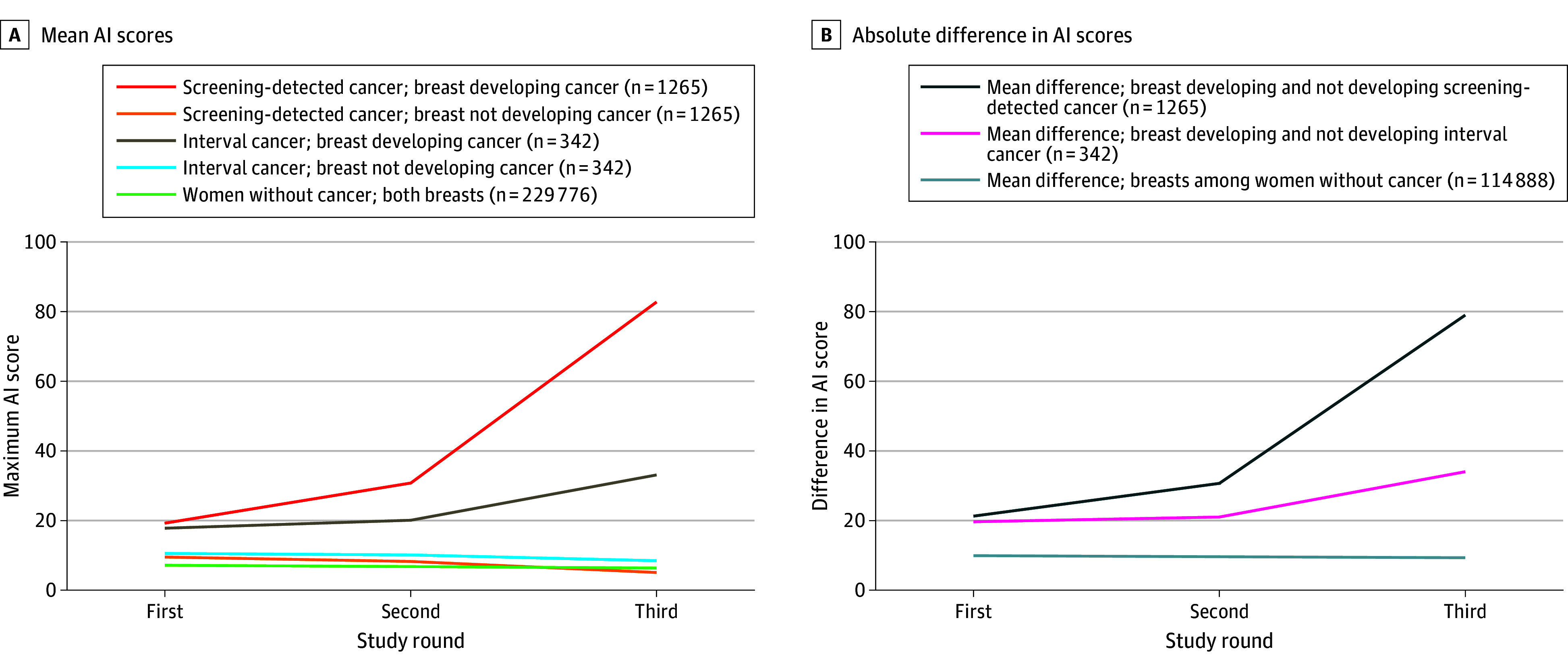
Mean Artificial Intelligence (AI) Scores and the Absolute Difference in AI Scores Between the Breasts for Each Study Round A, Mean AI scores for breasts not developing (negative) and developing screening-detected cancer and/or interval cancer and the mean of both breasts among women negatively screened in first, second, and third study screening rounds. B, Absolute difference in AI scores between the breasts for each study round.

The mean (SD) AI scores for the breast developing screening-detected cancer were 19.2 (28.6) at the first study round, 30.8 (34.4) at the second study round, and 82.7 (26.7) at the third study round (Table 1 and Figure 2). In comparison, for the breast not developing breast cancer, the mean (SD) AI scores were 9.5 (19.0) at the first study round, 8.2 (17.7) at the second study round, and 5.0 (15.7) at the third study round. For women with interval cancers, the mean (SD) AI scores for the breast developing cancer were 17.8 (26.3) at the first study round, 20.1 (27.3) at the second study round, and 33.1 (33.8) at the third study round. The mean (SD) AI scores for the contralateral breast not developing interval breast cancer were 10.5 (19.9) at the first study round, 10.1 (19.5) at the second study round, and 8.4 (18.7) at the third study round. Women with no breast cancer diagnosis during the study period had mean (SD) AI scores of 7.1 (15.2) at the first study round, 6.7 (14.9) at the second study round, and 6.4 (14.5) at the third study round. The AI scores increased by study round for both the CC and MLO images of the breast that developed cancer (eTable 2 in Supplement 1).

**Table 1.** AI Scores Given for the Breast That Developed and the Breast That Did Not Develop Screening-Detected or Interval Cancer and the Mean Across Both Breasts Among Women With No Breast Cancer

Cancer	Study round		
	First	Second	Third
**Screening-detected cancer**			
Breast developing breast cancer (n = 1265)			
AI score, mean (SD)	19.2 (28.6)	30.8 (34.4)	82.7 (26.7)
AI score, median (IQR)	3.7 (0.6-26.1)	12.3 (1.5-60.2)	96.4 (81.0-99.0)
Contralateral breast of that developing breast cancer (n = 1265)			
AI score, mean (SD)	9.5 (19.0)	8.2 (17.7)	5.0 (15.7)
AI score, median (IQR)	1.2 (0.3-7.4)	0.8 (0.1-5.9)	0.1 (0.0-1.1)
**Interval cancer**			
Breast developing breast cancer (n = 342)			
AI score, mean (SD)	17.8 (26.3)	20.1 (27.3)	33.1 (33.8)
AI score, median (IQR)	5.2 (1.0-21.4)	7.1 (0.9-30.1)	16.9 (3.3-56.0)
Contralateral breast of that developing breast cancer (n = 342)			
AI score, mean (SD)	10.5 (19.9)	10.1 (19.5)	8.4 (18.7)
AI score, median (IQR)	2.1 (0.5-8.7)	1.6 (0.4-8.7)	1.2 (0.3-5.5)
**Screening-detected cancer and interval cancer**			
Breast developing breast cancer (n = 1607)			
AI score, mean (SD)	18.9 (28.1)	28.5 (33.3)	72.2 (34.9)
AI score, median (IQR)	4.0 (0.6-25.4)	10.4 (1.4-54.5)	92.6 (45.2-98.7)
Contralateral breast of that developing breast cancer (n = 1607)			
AI score, mean (SD)	9.7 (19.2)	8.6 (18.1)	5.8 (16.4)
AI score, median (IQR)	1.4 (0.3-7.7)	1.0 (0.2-6.4)	0.2 (0.0-2.2)
**Examinations with negative screening results**			
Both breasts (n = 229 776)			
AI score, mean (SD)	7.1(15.2)	6.7 (14.9)	6.4 (14.5)
AI score, median (IQR)	1.1 (0.2-5.6)	0.9 (0.2-4.9)	0.8 (0.2-4.4)

Abbreviation: AI, artificial intelligence.

For women who developed screening-detected cancer, the mean (SD) absolute differences in AI score between breasts were 21.3 (28.1) at the first study round, 30.7 (32.5) at the second study round, and 79.0 (28.9) at the third study round (Table 2 and Figure 2). For women who developed an interval cancer, the mean (SD) absolute differences in AI score were 19.7 (27.0) at the first study round, 21.0 (27.7) at the second study round, and 34.0 (33.6) at the third study round. For women who did not develop breast cancer, the mean (SD) absolute differences in AI scores were 9.9 (17.5) at the first study round, 9.6 (17.4) at the second study round, and 9.3 (17.3) at the third study round.

**Table 2.** Absolute Differences in AI Scores Between Women Who Developed Screening-Detected or Interval Cancer and Women With No Breast Cancer

Cancer	Study round		
	First	Second	Third
**Screening-detected cancer (n = 1265)**			
Difference, mean (SD)	21.3 (28.1)	30.7 (32.5)	79.0 (28.9)
Difference, median (IQR)	6.6 (1.2-32.0)	16.2 (2.7-55.5)	94.3 (70.4-98.6)
**Interval cancer (n = 342)**			
Difference, mean (SD)	19.7 (27.0)	21.0 (27.7)	34.0 (33.6)
Difference, median (IQR)	6.1 (1.7-30.4)	8.2 (1.5-30.9)	19.8 (4.5-60.5)
**Screening-detected cancer and interval cancer (n = 1607)**			
Difference, mean (SD)	21.0 (27.8)	28.6 (31.8)	69.4 (35.1)
Difference, median (IQR)	6.4 (1.3-31.6)	13.7 (2.3-50.3)	88.7 (39.6-98.1)
**Examinations with negative screening results (n = 114 888)**			
Difference, mean (SD)	9.9 (17.5)	9.6 (17.4)	9.3 (17.3)
Difference, median (IQR)	2.2 (0.4-10.1)	2.0 (0.4-9.6)	1.8 (0.4-9.0)

Abbreviation: AI, artificial intelligence.

With the examination-level AI score, the AUCs for discriminating between women who developed screening-detected cancer and women with no cancer were 0.64 (95% CI, 0.62-0.65) at the first study round, 0.73 (95% CI, 0.71-0.74) at the second study round, and 0.97 (95% CI, 0.96-0.97) at the third study rounds (Table 3). The AUCs for interval cancers vs no cancer increased from 0.66 to 0.78 across the 3 study rounds. The AUCs for all cancers combined vs no cancer increased from 0.64 to 0.93 across the 3 study rounds. The AUCs for the absolute difference were 0.63 (95% CI, 0.61-0.65) at the first study round, 0.72 (95% CI, 0.71-0.74) at the second study round, and 0.96 (95% CI, 0.95-0.96) at the third study round for screening-detected cancer and 0.64 (95% CI, 0.61-0.67) at the first study round, 0.65 (95% CI, 0.62-0.68) at the second study round, and 0.77 (95% CI, 0.74-0.79) at the third study round for interval cancers.

**Table 3.** AUCs for Estimating Screening-Detected Cancer and Interval Cancer vs No Cancer by Screening Study Round

Cancer	Study round		
	First	Second	Third
**AUC (95% CI)**			
SDC	0.64 (0.62-0.65)	0.73 (0.71-0.74)	0.97 (0.96-0.97)
IC	0.66 (0.63-0.69)	0.68 (0.65-0.71)	0.78 (0.76-0.81)
SDC and IC	0.64 (0.63-0.66)	0.72 (0.70-0.73)	0.93 (0.92-0.93)
**Absolute difference (95% CI)**			
SDC	0.63 (0.61-0.65)	0.72 (0.71-0.74)	0.96 (0.95-0.96)
IC	0.64 (0.61-0.67)	0.65 (0.62-0.68)	0.77 (0.74-0.79)
SDC and IC	0.63 (0.62-0.65)	0.71 (0.69-0.72)	0.92 (0.91-0.93)

Abbreviations: AUC, area under the receiver operating characteristic curve; IC, interval cancer; SDC, screening-detected cancer.

If we defined the highest 1% of examination-level AI scores as positive and the other 99% as negative, representing an absolute AI score threshold of 91.3, 4.5% (73 of 1607) of cancers (screening-detected and interval) at the first study round, 8.6% (139 of 1607) of cancers at the second study round, and 52.9% (850 of 1607) of cancers at the third study round would have positive AI scores (eTable 3 in Supplement 1). At this same threshold, 0.7% of women in each study round (first: 849 of 114 888, second: 790 of 114 888, and third: 792 of 114 888) not developing breast cancer would have a false-positive AI score. Similar patterns were observed for hypothetical thresholds based on the differences in AI scores between breasts.

## Discussion

In this retrospective population-based cohort study involving multiple rounds of routine mammography in a national screening program, we found that a cancer detection AI algorithm was able to estimate future breast cancer based on a mammography performed 4 to 6 years before diagnosis. Although current commercial AI tools, such as the one used in our study, were not developed or optimized for future cancer risk estimations, we found that the AI system’s discriminatory accuracy for estimating future screening-detected or interval cancer risk 4 to 6 years prior to diagnosis met or exceeded the performance of established risk calculators currently in wide use, such as the Tyrer-Cuzick model (also known as IBIS [International Breast Intervention Study]), the Breast Cancer Risk Assessment Tool (BCRAT), and the Breast Cancer Surveillance Consortium model.^23,24,25,26,27^ The examination-level score and the difference in scores between the 2 breasts performed equally well. Future studies should also evaluate the potential incremental improvement in risk prediction when combining well-known clinical risk factors with AI scores.

To date, clinical risk factor–based models have demonstrated modest discrimination between women who do and women who do not develop cancer, with AUCs of 0.62 to 0.71 for the Tyrer-Cuzick model, 0.56 to 0.68 for the BCRAT, and 0.64 to 0.69 for the Breast Cancer Surveillance Consortium.^27,28^ The AUCs for the score of the AI tool that we tested for estimating screening-detected or interval cancers ranged from 0.64 to 0.73 for mammography 2 to 4 years prior to diagnosis of a screening-detected cancer and increased for interval cancers from 0.66 to 0.78 across all 3 consecutive screening rounds. The AUC for cancers diagnosed at or within 2 years of the third screening round was 0.93. Information about common risk factors for breast cancer is usually not available to radiologists during the interpretation of the screening mammography. An AI system that indicates the woman’s individual risk for breast cancer based solely on mammograms could provide a streamlined, more efficient approach to risk-based screening decisions if image-based AI is found to be as accurate as, or more accurate than, existing risk calculators.

In our study, AI scores were higher and increased more rapidly over the 3 successive screening rounds for women with a diagnosis of a screening-detected cancer vs an interval cancer. This finding suggests that interval cancers develop faster and may be less likely to show suspicious features on screening mammograms compared with screening-detected cancers, indicating that many interval cancers are truly mammographically occult at the time of screening and may not be detectable by the interpreting radiologists.^29,30,31^

Our large cohort study helps corroborate earlier, smaller studies suggesting that AI for cancer detection can also be used for future cancer risk estimation. One paired case-control study included 3386 women with a prior examination 3 years before cancer diagnosis to apply AI cancer detection algorithms for breast cancer risk estimation concluded that AI algorithms performing well for cancer detection also could be used for risk estimation.^15^ Another study including 1602 cancer cases (1016 screening-detected cancers and 586 interval cancers) showed that 39% of the cancer cases within the 10% highest AI cancer detection scores also were within the 10% highest scores 2 years before cancer diagnosis and that 23% were within the 10% highest scores 4 years before diagnosis.^31^

In our study, most future breast cancers were diagnosed in breasts with an examination-level AI score of higher than 20 and a difference in scores between breasts of higher than 20. The increasing difference in AI scores by time and between the breasts could be used by interpreting radiologists to indicate elevated risk of developing breast cancer and to suggest supplemental screening or annual mammography screening. Developing thresholds for risk estimation may help identify women who may benefit from more intensive mammography or supplemental screening.^32,33^

### Strengths and Limitations

Our retrospective population-based study has multiple strengths. First, unlike most prior reports, we have histologically verified breast cancer diagnoses with linkage to our national tumor registry, not relying on radiologist assessments or facility-based biopsy results that may underestimate interval cancers. Second, we used a regulatory-approved AI tool that is widely available, making our results more generalizable.

This study also has limitations. It was based on retrospective data, including screening-detected cancers and interval cancers detected by radiologists. This hampered evaluation of the real performance of an AI system as a stand-alone method. We evaluated 1 commercial AI system for cancer detection, but several others are currently available. Our study population tends to be homogeneous, with mostly White women. Further studies should evaluate the predictive accuracy of future cancer risk using other AI cancer detection tools and among more racially and ethnically diverse populations. As a result of our cohort selection, women with breast cancer were slightly older at inclusion and thus at higher risk of breast cancer. We consider the difference minor given the age range in the study. Older women might also have more examinations from earlier years than those without breast cancer. Similar systems were used throughout the study period, and any changes in image quality over time were thus considered limited. Finally, mammographic density might be higher among the women with breast cancer, which could have affected AI risk scores.^34,35^

## Conclusions

Our future research will include evaluating the location of markings flagged by the AI cancer detection tool and examining images interpreted as negative by radiologists but are in women who eventually have breast cancers diagnosed to determine if AI markings can better aid in guiding targeted supplemental screening. In addition, risk estimation models could be developed based on AI scores and differences in the AI scores for the 2 breasts over time and other factors, such as age, mammographic density, and prior benign breast biopsies, to provide an individual risk estimation for women regularly receiving mammography screening.^32,33,36^ Performance indicators such as sensitivity and specificity have not been reported but can be estimated based on the numbers given in eTable 2 in Supplement 1.

This cohort study found that, among women regularly undergoing mammography screening, AI cancer detection scores were elevated in the breast that developed breast cancer based on the screening mammography performed 4 to 6 years before their detection. Commercial AI algorithms may identify women at high risk of a future breast cancer diagnosis, offering a pathway for risk-based screening to promote earlier detection. Future use of AI for risk prediction to suggest additional imaging will need to address an acceptable rate of false positives resulting from AI-driven supplemental screening.

## Notes

Supplement 1. **eTable 1.** Number (n) of Women Developing Breast Cancer and Number and Percentage (n, %) of Breasts Developing Cancer With Higher AI Score Than the Breast not Developing Cancer During the Screening Study Rounds, Among all the Cancer Cases **eTable 2.** Mean With Standard Deviation (SD) and Median With Interquartile Range (IQR) Value of AI Score Given for the Breast That Developed and Did Not Develop Screen-Detected or Interval Cancer, and for Both Breast Among Those Not Developing Breast Cancer Given for Each Mammography View at Three Biennial Consecutive Screening Study Rounds in BreastScreen Norway **eTable 3.** Number (n) and Percentage (%) of Screen-Detected (SDC, n=1265), Interval Cancers (IC, n=342) and SDC+IC Combined (n=1607), and Negative Examinations (False Positive) by AI Score Thresholds and Absolute Difference in AI Score (Positive; Negative) Among 116 495 Screening Examinations

Supplement 2.
Data Sharing Statement
