# Supplementary material for: Artificial Intelligence Algorithm for Subclinical Breast Cancer Detection
Source: JAMA Netw Open. 2024 Oct 3;7(10):e2437402. doi: 10.1001/jamanetworkopen.2024.37402 (PMC11450515; doi:10.1001/jamanetworkopen.2024.37402)
Supplement: Supplement 1. — eTable 1. Number (n) of Women Developing Breast Cancer and Number and Percentage (n, %) of Breasts Developing Cancer With Higher AI Score Than the Breast not Developing Cancer During the Screening Study Rounds, Among all the Cancer Cases eTable 2. Mean With Standard Deviation (SD) and Median With Interquartile Range (IQR) Value of AI Score Given for the Breast That Developed and Did Not Develop Screen-Detected or Interval Cancer, and for Both Breast Among Those Not Developing Breast Cancer Given for Each Mammography View at Three Biennial Consecutive Screening Study Rounds in BreastScreen Norway eTable 3. Number (n) and Percentage (%) of Screen-Detected (SDC, n=1265), Interval Cancers (IC, n=342) and SDC+IC Combined (n=1607), and Negative Examinations (False Positive) by AI Score Thresholds and Absolute Difference in AI Score (Positive; Negative) Among 116 495 Screening Examinations [file jamanetwopen-e2437402-s001.pdf]

Supplementary Online Content

Gjesvik J, Moshina N, Lee CI, Miglioretti DL, Hofvind S. Artificial intelligence algorithm for subclinical breast cancer detection. *JAMA Netw Open.* 2024;7(10):e2437402. doi:10.1001/jamanetworkopen.2024.37402

eTable 1. Number (n) of Women Developing Breast Cancer and Number and Percentage (n, %) of Breasts Developing Cancer With Higher AI Score Than the Breast not Developing Cancer During the Screening Study Rounds, Among all the Cancer Cases
eTable 2. Mean With Standard Deviation (SD) and Median With Interquartile Range (IQR) Value of AI Score Given for the Breast That Developed and Did Not Develop Screen-Detected or Interval Cancer, and for Both Breast Among Those Not Developing Breast Cancer Given for Each Mammography View at Three Biennial Consecutive Screening Study Rounds in BreastScreen Norway
eTable 3. Number (n) and Percentage (%) of Screen-Detected (SDC, n=1265), Interval Cancers (IC, n=342) and SDC+IC Combined (n=1607), and Negative Examinations (False Positive) by AI Score Thresholds and Absolute Difference in AI Score (Positive; Negative) Among 116 495 Screening Examinations

This supplementary material has been provided by the authors to give readers additional information about their work.

eTable 1. Number (n) of Women Developing Breast Cancer and Number and Percentage (n, %) of Breasts Developing Cancer With Higher AI Score Than the Breast not Developing Cancer During the Screening Study Rounds, Among all the Cancer Cases

Screen-detected cancer	Women developing screen-detected cancer (n)	Breast developing screen-detected breast cancer with an AI score value >20 higher than the contralateral breast without cancer (n, %)	
First study round	1 265	301	24 %
Second study round	1 265	496	39 %
Third study round	1265	1 150	91 %
Interval cancer	Women developing interval cancer (n)	Breast developing interval breast cancer with an AI score value >20 higher than the contralateral breast without cancer (n, %)	
First study round	342	67	20 %
Second study round	342	79	23 %
Third study round	342	147	43 %
Screen-detected and interval cancer	Women developing screen-detected or interval cancer (n)	Breast developing screen-detected or interval breast cancer with an AI score value >20 higher than the contralateral breast without cancer (n, %)	
First study round	1607	368	23 %
Second study round	1607	575	36 %
Third study round	1607	1297	81 %
No cancer	Women not developing cancer (n)	One breast (randomly chosen) with an AI score value >20 higher than the other breast (n, %)	
First study round	114 888	8 907	8 %
Second study round	114 888	8 544	7 %
Third study round	114 888	8 237	7 %

© 2024 Gjesvik J et al. *JAMA Network Open.*

eTable 2. Mean With Standard Deviation (SD) and Median With Interquartile Range (IQR) Value of AI Score Given for the Breast That Developed and Did Not Develop Screen-Detected or Interval Cancer, and for Both Breast Among Those Not Developing Breast Cancer Given for Each Mammography View at Three Biennial Consecutive Screening Study Rounds in BreastScreen Norway

	First study round			Second study round			Third study round		
	CC	MLO	CC and MLO	CC	MLO	CC and MLO	CC	MLO	CC and MLO
Screen-detected cancer									
The breast developing breast cancer (n = 1,265)									
AI Mean score (SD)	15.4 (25.6)	15.7 (25.9)	19.2 (28.6)	25.5 (31.8)	24.2 (31.2)	30.8 (34.4)	75.6 (31.3)	72.0 (32.6)	82.7 (26.7)
AI Median score (IQR)	2.2 (0.4 - 17.4)	2.3 (0.4 - 18.0)	3.7 (0.6 - 26.1)	7.6 (1.0 - 44.9)	6.6 (0.9 - 42.3)	12.3 (1.5 - 60.2)	92.7 (58.2 - 98.5)	89.0 (47.8 - 98.5)	96.4 (81.0 - 99.0)
The contralateral breast of that developing breast cancer (n = 1,265)									
AI Mean score (SD)	7.2 (16.1)	7.6 (17.0)	9.5 (19.0)	6.2 (15.3)	6.5 (15.9)	8.2 (17.7)	4.2 (14.4)	4.4 (14.5)	5.0 (15.7)
AI Median score (IQR)	0.9 (0.2 - 5.1)	0.8 (0.2 - 4.4)	1.2 (0.3 - 7.4)	0.5 (0.1 - 3.6)	0.5 (0.1 - 3.4)	0.8 (0.1 - 5.9)	0.1 (0.0 - 0.7)	0.1 (0.0 - 0.8)	0.1 (0.0 - 1.1)
Interval cancer									
The breast developing breast cancer (n = 342)									
AI Mean score (SD)	14.8 (24.4)	14.8 (23.9)	17.8 (26.3)	16.2 (24.3)	16.4 (25.2)	20.1 (27.3)	28.1 (31.8)	26.1 (30.9)	33.1 (33.8)
AI Median score (IQR)	3.3 (0.7 - 16.3)	3.5 (0.6 - 15.7)	5.2 (1.0 - 21.4)	4.0 (0.5 - 20.2)	4.0 (0.5 - 20.2)	7.1 (0.9 - 30.1)	13.1 (2.3 - 45.7)	11.7 (1.9 - 39.5)	16.9 (3.3 - 56.0)
The contralateral breast of that developing breast cancer (n = 342)									
AI Mean score (SD)	8.2 (17.1)	8.4 (18.2)	10.5 (19.9)	8.0 (17.4)	8.2 (17.5)	10.1 (19.5)	7.2 (17.6)	6.7 (16.4)	8.4 (18.7)
AI Median score (IQR)	1.5 (0.3 - 6.0)	1.3 (0.3 - 6.0)	2.1 (0.5 - 8.7)	1.2 (0.3 - 6.4)	1.3 (0.3 - 5.4)	1.6 (0.4 - 8.7)	0.8 (0.2 - 3.5)	0.8 (0.2 - 4.5)	1.2 (0.3 - 5.5)
Screen-detected and interval cancer									
The breast developing breast cancer (n = 1,607)									
AI Mean score (SD)	15.3 (25.3)	15.5 (25.5)	18.9 (28.1)	23.5 (30.6)	22.5 (30.2)	28.5 (33.3)	65.5 (36.9)	62.2 (37.3)	72.2 (34.9)
AI Median score (IQR)	2.6 (0.4 - 17.5)	2.6 (0.4 - 17.5)	4.0 (0.6 - 25.4)	6.7 (0.9 - 39.2)	5.9 (0.8 - 36.4)	10.4 (1.4 - 54.5)	85.3 (29.8 - 97.9)	77.5 (24.7 - 97.5)	92.6 (45.2 - 98.7)
The contralateral breast of that developing breast cancer (n = 1,607)									
AI Mean score (SD)	7.4 (16.3)	7.8 (17.3)	9.7 (19.2)	6.6 (15.8)	6.9 (16.2)	8.6 (18.1)	4.8 (15.2)	4.9 (15.0)	5.8 (16.4)

© 2024 Gjesvik J et al. *JAMA Network Open.*

AI Median score (IQR)	0.9 (0.2 - 5.4)	0.9 (0.2 - 4.8)	1.4 (0.3 - 7.7)	0.6 (0.1 - 4.0)	0.6 (0.1 - 3.9)	1.0 (0.2 - 6.5)	0.1 (0.0 - 1.2)	0.1 (0.0 - 1.4)	0.2 (0.0 - 2.2)
Examinations with negative screening results									
Left breast (n = 114,888)									
AI Mean score (SD)	5.5 (13.0)	5.4 (13.0)	7.2 (15.1)	5.1 (12.6)	5.2 (12.7)	6.8 (14.8)	4.8 (12.3)	4.9 (12.4)	6.5 (14.5)
AI Median score (IQR)	0.7 (0.2 - 3.8)	0.7 (0.2 - 3.6)	1.1 (0.2 - 5.7)	0.6 (0.1 - 3.3)	0.6 (0.1 - 3.3)	1.0 (0.2 - 5.2)	0.6 (0.1 - 3.0)	0.6 (0.1 - 2.9)	0.9 (0.2 - 4.6)
Right breast (n = 114,888)									
AI Mean score (SD)	5.3 (12.8)	5.5 (13.3)	7.1 (15.2)	4.9 (12.5)	5.1 (12.9)	6.6 (14.9)	4.6 (12.3)	4.8 (12.6)	6.3 (14.6)
AI Median score (IQR)	0.7 (0.2 - 3.5)	0.7 (0.2 - 3.6)	1.1 (0.2 - 5.5)	0.6 (0.1 - 3.0)	0.6 (0.1 - 3.0)	0.9 (0.2 - 4.7)	0.5 (0.1 - 2.6)	0.5 (0.1 - 2.7)	0.8 (0.2 - 4.2)

© 2024 Gjesvik J et al. *JAMA Network Open.*

eTable 3. Number (n) and Percentage (%) of Screen-Detected (SDC, n=1265), Interval Cancers (IC, n=342) and SDC+IC Combined (n=1607), and Negative Examinations (False Positive) by AI Score Thresholds and Absolute Difference in AI Score (Positive; Negative) Among 116 495 Screening Examinations

Basis for the thresholds	First Study Round		Second Study Round		Third Study Round	
Screen-detected cancer<br>AI Score Threshold; % positive	SDC<br>n (%)	False positive<br>n (%)	SDC<br>n (%)	False positive<br>n (%)	SDC<br>n (%)	False positive<br>n (%)
≥ 91.3; 1%	55 (4.3%)	867 (0.8%)	123 (9.7%)	806 (0.7%)	809 (64.0%)	833 (0.7%)
≥ 60.8; 5%	222 (17.5%)	5 507 (4.8%)	343 (27.1%)	5 294 (4.6%)	1 060 (83.8%)	5 046 (4.4%)
≥ 38.3; 10%	337 (26.6%)	11 561 (10.0%)	483 (38.2%)	10 975 (9.5%)	1 133 (89.6%)	10 456 (9.1%)
≥ 18.0; 20%	502 (39.7%)	23 708 (20.6%)	669 (52.9%)	22 428 (19.5%)	1 199 (94.8%)	21 379 (18.6%)
≥ 9.6; 30%	636 (50.3%)	35 719 (31.0%)	784 (62.0%)	34 005 (29.5%)	1 229 (97.2%)	32 430 (28.1%)
≥ 3.1; 50%	865 (68.4%)	59 537 (51.7%)	984 (77.8%)	56 926 (49.4%)	1 253 (99.1%)	55 117 (47.8%)
Difference in AI Score Threshold: % positive						
≥ 87.7; 1%	64 (5.1%)	826 (0.7%)	124 (9.8%)	812 (0.7%)	783 (61.9%)	885 (0.8%)
≥ 53.4: 5%	207 (16.4%)	5 410 (4.7%)	333 (26.3%)	5 266 (4.6%)	1 033 (81.7%)	5 222 (4.5%)
≥ 32.0; 10%	315 (24.9%)	11 391 (9.9%)	468 (37.0%)	11 016 (9.6%)	1 111 (87.8%)	10 644 (9.2%)
≥ 14.2; 20%	487 (38.5%)	23 236 (20.2%)	666 (52.6%)	22 521 (19.5%)	1 181 (93.4%)	21 800 (18.9%)
≥ 7.1; 30%	618 (48.9%)	35 273 (30.6%)	791 (62.5%)	34 080 (29.6%)	1 215 (96.0%)	32 868 (28.5%)
≥ 2.1; 50%	861 (68.1%)	59 106 (51.3%)	975 (77.1%)	56 959 (49.4%)	1 245 (98.4%)	55 543 (48.2%)
Interval cancer<br>AI Score Threshold; % positive	IC<br>n (%)	False positive<br>n (%)	IC<br>n (%)	False positive<br>n (%)	IC<br>n (%)	False positive<br>n (%)
≥ 91.3; 1%	18 (5.3%)	849 (0.7%)	16 (4.7%)	790 (0.7%)	41 (12.0%)	792 (0.7%)
≥ 60.8; 5%	50 (14.6%)	5 457 (4.7%)	55 (16.1%)	5 239 (4.6%)	91 (26.6%)	4 955 (4.3%)
≥ 38.3; 10%	83 (24.3%)	11 478 (10.0%)	91 (26.6%)	10 884 (9.5%)	143 (41.8%)	10 313 (9.0%)
≥ 18.0; 20%	134 (39.2%)	23 574 (20.5%)	142 (41.5%)	22 286 (19.4%)	195 (57.0%)	21 184 (18.4%)
≥ 9.6; 30%	170 (49.7%)	35 549 (30.9%)	189 (55.3%)	33 816 (29.4%)	245 (71.6%)	32 185 (28.0%)
≥ 3.1; 50%	255 (74.6%)	59 282 (51.6%)	251 (73.4%)	56 674 (49.3%)	289 (84.5%)	54 828 (47.7%)

© 2024 Gjesvik J et al. *JAMA Network Open.*

Difference in AI Score Threshold: % positive						
≥ 87.7; 1%	14 (4.1%)	812 (0.7%)	17 (5.0%)	795 (0.7%)	43 (12.6%)	842 (0.7%)
≥ 53.4: 5%	47 (13.7%)	5 363 (4.7%)	50 (14.6%)	5 216 (4.5%)	93 (27.2%)	5 129 (4.5%)
≥ 32.0; 10%	80 (23.4%)	11 311 (9.8%)	82 (24.0%)	10 934 (9.5%)	142 (41.5%)	10 502 (9.1%)
≥ 14.2; 20%	120 (35.1%)	23 116 (20.1%)	129 (37.7%)	22 392 (19.5%)	200 (58.5%)	21 600 (18.8%)
≥ 7.1; 30%	163 (47.7%)	35 110 (30.6%)	179 (52.3%)	33 901 (29.5%)	234 (68.4%)	32 634 (28.4%)
≥ 2.1; 50%	247 (72.2%)	58 859 (51.2%)	241 (70.5%)	56 718 (49.4%)	282 (82.5%)	55 261 (48.1%)
Screen-detected and interval cancer<br>AI Score Threshold; % positive	SDC + IC<br>n (%)	False positive<br>n (%)	SDC + IC<br>n (%)	False positive<br>n (%)	SDC + IC<br>n (%)	False positive<br>n (%)
≥ 91.3; 1%	73 (4.5%)	849 (0.7%)	139 (8.6%)	790 (0.7%)	850 (52.9%)	792 (0.7%)
≥ 60.8; 5%	272 (16.9%)	5 457 (4.7%)	398 (24.8%)	5 239 (4.6%)	1 151 (71.6%)	4 955 (4.3%)
≥ 38.3; 10%	420 (26.1%)	11 478 (10.0%)	574 (35.7%)	10 884 (9.5%)	1 276 (79.4%)	10 313 (9.0%)
≥ 18.0; 20%	636 (39.6%)	23 574 (20.5%)	811 (50.5%)	22 286 (19.4%)	1 394 (86.7%)	21 184 (18.4%)
≥ 9.6; 30%	802 (49.9%)	35 549 (30.9%)	973 (60.5%)	33 816 (29.4%)	1 474 (91.7%)	32 185 (28.0%)
≥ 3.1; 50%	1 120 (69.7%)	59 282 (51.6%)	1 235 (76.9%)	56 675 (49.3%)	1 542 (96.0%)	54 828 (47.7%)
Difference in AI Score Threshold: % positive						
≥ 87.7; 1%	78 (4.9%)	812 (0.7%)	141 (8.8%)	795 (0.7%)	826 (51.4%)	842 (0.7%)
≥ 53.4: 5%	254 (15.8%)	5 363 (4.7%)	383 (23.8%)	5 216 (4.5%)	1 126 (70.1%)	5 129 (4.5%)
≥ 32.0; 10%	395 (24.6%)	11 311 (9.8%)	550 (34.2%)	10 934 (9.5%)	1 253 (78.0%)	10 502 (9.1%)
≥ 14.2; 20%	607 (37.8%)	23 116 (20.1%)	795 (49.5%)	22 392 (19.5%)	1 381 (85.9%)	21 600 (18.8%)
≥ 7.1; 30%	781 (48.6%)	35 110 (30.6%)	970 (60.4%)	33 901 (29.5%)	1 449 (90.2%)	32 634 (28.4%)
≥ 2.1; 50%	1 108 (68.9%)	58 859 (51.2%)	1 216 (75.7%)	56 718 (49.4%)	1 527 (95.0%)	55 261 (48.1%)

© 2024 Gjesvik J et al. *JAMA Network Open.*
