# Supplementary material for: Artificial Intelligence Algorithm for Subclinical Breast Cancer Detection
Source: JAMA Netw Open. 2024 Oct 3;7(10):e2437402. doi: 10.1001/jamanetworkopen.2024.37402 (PMC11450515; doi:10.1001/jamanetworkopen.2024.37402)
Supplement: Supplement 2. — Data Sharing Statement [file jamanetwopen-e2437402-s002.pdf]

Data Sharing Statement

Gjesvik. Artificial Intelligence Algorithm for Subclinical Breast Cancer Detection. *JAMA Netw Open*. Published October 03, 2024. doi:10.1001/jamanetworkopen.2024.37402

Data
Data available: No

Additional Information
Explanation for why data not available: Research data used in the analyses can be made available on request to https://helsedata.no/, given legal basis in Articles 6 and 9 of the GDPR and that the processing is in accordance with Article 5 of the GDPR.
